# Bestimmungsfaktoren für landwirtschaftliche Bewässerungsbedarfe und regionale Governance-Ansätze zur Konfliktreduktion in Niedersachsen und Sachsen-Anhalt

**DOI:** 10.1007/s00767-023-00543-8

**Published:** 2023-05-09

**Authors:** Elisabeth Schulz, Christina Scharun

**Affiliations:** 1 Bezirksstelle Uelzen, Landwirtschaftskammer Niedersachsen, 29525 Uelzen, Wilhelm-Seedorf-Str. 3, Deutschland; 2 Referat für Bodenschutz, Bodenkundliche Landesaufnahme, Landesamt für Bergbau, Energie und Geologie, Stilleweg 2, 30655 Hannover, Deutschland

**Keywords:** Klimawandelauswirkungen, Wassermangel, Potentielle Bewässerungsbedarfe, Governance, Reibungsverluste, Regionale Stakeholder-Netzwerke, Vertrauensbildung, Anpassungsmaßnahmen, Climate change impacts, Water shortage, Potential irrigation demand, Governance, Friction losses, Regional stakeholder networks, Confidence building, Adaptation measures

## Abstract

Zunehmende Trockenheit erhöht landwirtschaftliche Bewässerungsbedarfe. Erstmalige Grundwasserentnahmeanträge sowie Erweiterungsanträge nehmen zu. Damit verändern sich langjährige Gleichgewichte unter den Grundwassernutzern (Trinkwassergewinnung, Ökosystemschutz, Gewässerunterhaltung, Forstwirtschaft, Landwirtschaft, Verwaltung, Öffentlichkeit). Widerstände gegen Behördenentscheidungen könnten anwachsen. Um diesbezüglich potenzielle ressourcenzehrende Reibungsverluste zu verringern und stattdessen kooperatives Handeln anzustoßen, erprobten Landwirtschaftskammer Niedersachen und Landesamt für Bergbau, Energie und Geologie mit ausgewählten Landkreisen in zwei Projekten (*Netzwerke Wasser)* neuartige governance-basierte Handlungsansätze. *Runde Tische* mit Stakeholdervertretern wurden eingerichtet zwecks Kennenlernens, Kompetenzerweiterung und Vertrauensbildung. Während ganztägiger „Netzwerktreffen“ wurden regionalspezifische Fachinformationen einschließlich der Bestimmungsfaktoren für landwirtschaftliche Wassernachfrage vermittelt. Insbesondere fehlten objektive Informationen über aktuelle sowie zukünftige landwirtschaftliche Bewässerungsbedarfe. Deshalb wurden die örtlichen potenziellen Beregnungsbedürftigkeiten auf Basis hochaufgelöster Bodendaten, Ackerkulturartenanteilen und Klima(projektions)daten ermittelt. Eindeutige Trends zu steigenden potenziellen Beregnungsbedarfen von bis zu 31 % im Gebietsmittel bis Jahrhundertende wurden festgestellt. Besonders wichtig waren die Möglichkeiten für informellen Austausch der Handelnden. Abschließend überwog deutlich der Wunsch nach Verstetigung der Stakeholder-Plattformen.

## Abstract (en)

Increasing drought induces growing water demands from crop farming. Subsequently, “traditional balances” among groundwater stakeholders shift, and opposition to administrative regulations becomes more likely. For counteracting resource-consuming intersectoral friction, two projects (“Water Networks”) focusing on improving governance approaches were realized in selected districts. Round tables of selected representatives of regional groundwater stakeholders (drinking water, environmental protection, waterbody maintenance, forestry, agricultural irrigation) were established to enhance competences and understanding and to build mutual confidence. During all-day meetings with breaks for informal exchange, experts presented regional information including determinants for agricultural water demand. In particular, objective information was found lacking on recent and future irrigation demands for crops. Thus, potential regional irrigation needs were determined based on high-resolution soil data, climate data and distribution of primary crops. Clear trends towards increasing requirements of irrigation of up to 31% in regional average were identified until the end of the century. The need for continuing the platform discussions was a main conclusion of the participants.

## Zusammenfassung

Die in den letzten Jahren wahrgenommene Häufung und Intensivierung von Trockenperioden führte – neben anderen Anpassungsmaßnahmen – zu einer gestiegenen Anzahl von Erlaubnisanträgen zur Grundwasserentnahme für die landwirtschaftliche Bewässerung (Feldberegnung). Dies betrifft zunehmend Regionen und deren Akteure, die zuvor nicht mit dem Thema befasst waren und deshalb wenig Kenntnis über die Situation der diversen regionalen wasserbezogenen Handlungsfelder sowie Stakeholder haben. Vor diesem Hintergrund entwickelten und erprobten die Landwirtschaftskammer Niedersachsen (LWK) und das niedersächsische Landesamt für Bergbau, Energie und Geologie (LBEG) in zwei aufeinander folgenden Projekten („Netzwerke Wasser“) eine Herangehensweise zur Vernetzung regionaler Wasser-Stakeholder, die hier vorgestellt wird. Die Finanzierung erfolgte mit Fördermitteln aus der Deutschen Anpassungsstrategie (DAS) zur Entwicklung von u. a. kommunalen Anpassungsstrategien. Das Ziel war, pilothaft in betroffenen Regionen mögliche kräftezehrende Reibungsverluste im Klimaanpassungsprozess zu vermeiden. Die beschriebenen Projektaktivitäten erfolgten unter der Hypothese, dass betroffene Regionen durch eine frühzeitige Vernetzung der Wasser-Stakeholder widerstandsfähiger gegen wasserwirtschaftliche Klimawirkungen sind. Als eine wesentliche Voraussetzung für eine effiziente Anpassung wurde die Kenntnis der aktuellen und zukünftigen regional-spezifischen Bewässerungsbedarfe gesehen. Die hier vorliegende Veröffentlichung erläutert die durchgeführten Arbeiten in Form eines Projektberichts. Zum weitergehenden Verständnis werden eingangs die Bestimmungsfaktoren für die Anwendung des landwirtschaftlichen Betriebsmittels Zusatzwasser sowie die allgemeinen Rahmenbedingungen der Feldberegnung dargestellt.

In den Projekten wurden mit dem Ziel der Vernetzung zur Schaffung von Kooperationsmöglichkeiten in ausgewählten Landkreisen Niedersachsens und Sachsen-Anhalts fünf regionale Runde Tische – die sogenannten „Netzwerke Wasser“ – eingerichtet und während zweimal drei Jahren betreut. Eine Fortsetzung in extensiverer Form wurde abschließend durch die Stakeholder vereinbart und vorbereitet. Die Netzwerk-Teilnehmenden identifizierten in regionalen Vulnerabilitätsanalysen die Verfügbarkeit von Wasser als einen wesentlichen zukünftigen Faktor ihrer Regionalentwicklung. Mit dem Ziel, exemplarisch die derzeitige planerische Situation der ausgewählten Landkreise zu verbessern, wurden für diese die regionalen Zusatzwasserbedarfe der dort jeweils wichtigsten Ackerkulturen ermittelt sowie die erwarteten Auswirkungen des Klimawandels darauf. Für letzteres wurden Methoden zur Bestimmung potenzieller Zusatzwasserbedarfe (siehe Kapitel „Methode der Abschätzung des potenziellen Beregnungsbedarfs unter Berücksichtigung des Klimawandels“) angewandt und mit Klimaprojektionsdaten verknüpft. Der Trend der Zunahme der potenziellen Beregnungsbedürftigkeit in der Zukunft im Emissionsszenario RCP8.5 des aktuellen IPCC-Berichts (Pachauri und Meyer 2014) ist aus den errechneten Daten konsistent über alle Netzwerkregionen ablesbar. Der Anstieg wird auf 10 bis 30 % bis 2100 modelliert. Des Weiteren wurde die länderübergreifende Übertragung der Methode am Beispiel von Sachsen-Anhalt erprobt.

Die Einrichtung und Betreuung der Netzwerke erfolgte maßgeblich durch die LWK in Kooperation mit den beteiligten Unteren Wasserbehörden. Die bodenkundlichen Planungsgrundlagen sowie wesentliche Beiträge zum fachlichen Input in die Netzwerke wurden durch das LBEG erarbeitet.

## Einleitung

Die Landwirtschaftskammer Niedersachsen (LWK) erlebte in den letzten zehn Jahren eine vergleichsweise sprunghaft gestiegene Nachfrage nach Beratung hinsichtlich eines möglichen Einstiegs landwirtschaftlicher Betriebe in die sogenannte „Feldberegnung“. Parallel berichteten regional betroffene Untere Wasserbehörden und das Landesamt für Bergbau, Energie und Geologie (LBEG, Hannover) von einem ungekannten Anstieg der Anzahl wasserrechtlicher Erstanträge für Bewässerung aus Grundwasser. Die räumlichen Schwerpunkte dieses Geschehens lagen außerhalb der traditionellen Beregnungsgebiete Niedersachsens sowie in benachbarten Bundesländern. Hintergründe hierfür sind u. a. der klimawandelbedingte Temperaturanstieg mit intensiverer Verdunstung sowie die in der landwirtschaftlichen Praxis als existenzbedrohend wahrgenommene Ausdehnung und zugleich Häufung von Trockenperioden während der Vegetationszeit. Auf den in Norddeutschland verbreitet vorkommenden grundwasserfernen Geest-Standorten mit geringem Wasserspeichervermögen wurden wiederholt teilweise erhebliche Ertragsrückgänge bei allen bedeutenden Ackerkulturen verzeichnet.

Neben dieser neuartigen Nachfrage nach Wasser für die Feldberegnung stiegen zugleich häufig auch die Wasserbedarfe anderer örtlicher Sektoren wie der Schutz grundwasserabhängiger Biotope oder die Bereitstellung von Trink- und Brauchwasser. Diese Veränderungen bewirken auch die Veränderung von bisherigen langjährigen Beziehungen und Wahrnehmungen unter den Grundwasser-Akteuren (Grundwasser-Stakeholdern) und bergen damit wesentliche Konfliktpotenziale. Erfahrungen oder Anschauungsmöglichkeiten für erfolgreiche Anpassungsaktivitäten in der Wasserwirtschaft an Knappheit fehlen bisher zumeist. Deshalb bestand seitens der projektbeteiligten Institutionen LWK, LBEG und Landkreise die Befürchtung von kräftezehrenden und damit den regionalen Wohlstand mindernden Reibungsverlusten. Folgende neuartige Herausforderungen werden erkennbar:

- Der Klimawandel verstärkt die ohnehin gegebene Komplexität des Themas „Wassernutzung“.
- Wasserwirtschaftliche Problemstellungen sowie Handlungsmöglichkeiten sind regional teilweise sehr verschieden.
- Das Thema „Wasser“ birgt eine beträchtliche Emotionalität.
- Das bisher ungekannte Tempo der Entwicklungen einschließlich drohender Verluste bzw. erforderlicher Anpassungsbedarfe erhöht die Gefahr von Konfrontationen zwischen den Akteuren.

In dem neuen Handlungsfeld Wasserverknappung treffen naturwissenschaftliche und technische Herausforderungen auf kommunikationsbezogene und emotionale Handlungsebenen. Die LWK und das LBEG gehen davon aus, dass die zukünftige Anpassung an diese Veränderungen am erfolgreichsten im Sinne des Gemeinwohls durch konstruktive Zusammenarbeit aller Wasserakteure einer Region erreicht werden kann. Ein Ansatz für ein entsprechendes praktisches Vorgehen auf Ebene regionaler Erlaubnisbehörden (Landkreise) wurde in zwei gemeinsamen Projekten von LWK und LBEG unter dem Titel „Netzwerke Wasser“ konzipiert und gemeinsam mit fünf Partnerlandkreisen umgesetzt. Im Fokus standen einerseits die Bereitstellung und Vermittlung regionalbezogener Sachinformationen und andererseits die Herstellung geeigneter regionaler Governance-Strukturen.

Auslöser der Netzwerke Wasser-Projekte war die beschriebene neuartige Nachfrage nach Beregnungswasser. Deshalb erfolgt nachfolgend vor dem eigentlichen Praxisbericht eine Darstellung der Bestimmungsfaktoren für einen Einstieg in die landwirtschaftliche Bewässerung in Deutschland sowie deren Rahmenbedingungen. Von besonderer Bedeutung für die Netzwerk-Teilnehmenden war eine neutrale Information über den aktuellen und den erwartbaren potenziellen Bewässerungsbedarf in ihrer Region. Deshalb werden die vom LBEG hierfür weiterentwickelte Methodik, die regionalen Resultate und die Diskussion in eigenen Abschnitten ausführlich dargestellt.

## Bestimmungsfaktoren für Investitionen in die landwirtschaftliche Bewässerung sowie für ihren anschließenden Einsatz

Die aus den fehlenden Niederschlägen während der Vegetationszeit zunehmend resultierenden Ertrags- und damit Einkommensverluste werden von vielen Landwirtinnen und Landwirten auf leichten Standorten als existenzbedrohend eingestuft. Grundsätzlich bestimmen – neben den allgemeinen wirtschaftlichen Rahmenbedingungen – die lokalen Standorteigenschaften Art und Ausprägung von Flächennutzungs- und Bewirtschaftungssystemen (Klima, Höhenlage, Böden, deren Wasserspeichervermögen i.e. nutzbare Feldkapazität im effektiven Wurzelraum (nFKWe), Grundwasserverhältnisse etc.). Synchron mit der Technisierung der Landwirtschaft wird seit Mitte des letzten Jahrhunderts bei trockenen Standortverhältnissen insbesondere in Niedersachsen und bis zur Grenzöffnung in der ehemaligen DDR ebenso wie in den meisten nord- und mitteleuropäischen Ländern zur Verbesserung des natürlichen Standortpotenzials, wenn möglich, Bewässerung in Form von Feldberegnung eingesetzt. Dabei wird im landwirtschaftlichen Kontext die sogenannte Beregnungs*bedürftigkeit* von der Beregnungs*würdigkeit* unterschieden. Unter Beregnungs*bedürftigkeit* wird der potenzielle Bedarf der Pflanze nach Zusatzwasser verstanden, der sich aus ihrer Physiologie und den Standorteigenschaften ergibt (siehe Kapitel „Methode der Abschätzung des potenziellen Beregnungsbedarfs unter Berücksichtigung des Klimawandels“). In den Begriff der Beregnungs*würdigkeit* fließen zusätzlich ökonomische Faktoren ein, die im Folgenden beschrieben werden.

Neben den natürlichen Rahmenbedingungen steht ein maßgeblicher ökonomischer Bestimmungsfaktor für oder gegen die Ausgestaltung als „Beregnungsbetrieb“, der selten Beachtung findet. Dies ist der üblicherweise deutlich über fünfzig Prozent liegende Pachtflächenanteil bei den sogenannten Zukunftsbetrieben (z. B. auf langfristige Bewirtschaftung ausgerichteten Betrieben). Denn die sehr starke Konkurrenz um landwirtschaftliche Flächen (Pacht, Erwerb) bewirkt, dass die höchsten Gebote erfolgreich sind. Um anschließend die Kosten des Erwerbs oder der Pacht zu erwirtschaften, ist eine entsprechende ackerbauliche Intensität erforderlich (z. B. Kulturarten mit hoher relativer Wertschöpfung inkl. Feldgemüse, Umstellung auf Biolandbau oder regionale Vermarktung, Ertragsniveau). Strategien einer ökonomischen Extensivierung des Ackerbaus – ohne finanziellen Ausgleich durch Dritte oder eine Betätigung als Hobby – sind regelmäßig ausgeschlossen.

**Figure 1:**
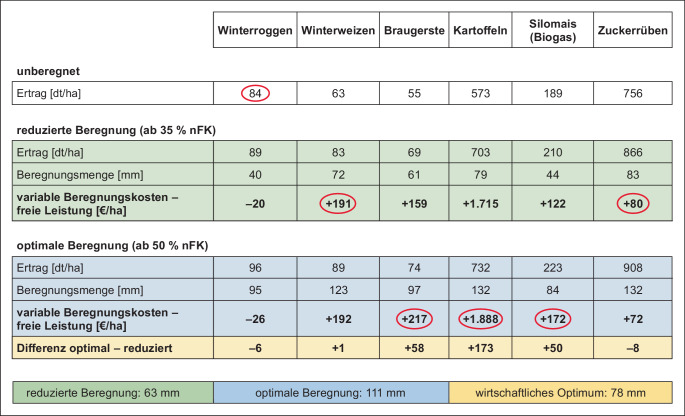

Ackerbau bietet als einzige Landnutzungsart die Möglichkeit einer jährlich neuen Anpassung der Fruchtart (z. B. Sommerung/Winterung), der Sorten (z. B. frühe/späte Ernte) und der Anbauverfahren (z. B. tiefe/flache/wendende/nicht wendende Bodenbearbeitung, Reihenabstände, Dammanbau, u. a. m.). Weil sich die Witterungsverläufe unterscheiden, wählen die Landwirte und Landwirtinnen auf Basis eigener Erfahrungen sowie überbetrieblicher Versuchsergebnisse (z. B. regionalisierte, fortlaufende Landessortenversuche) eine für ihre Flächen im Mittel der Jahre geeignete Strategie. Unter Berücksichtigung der phytosanitären und marktwirtschaftlichen Anforderungen resultiert so eine optimale Anpassung an die spezifischen örtlichen Standortbedingungen inklusive Wasserverfügbarkeit. Beispielhaft werden in der Tabelle in Abb. 1 die jeweils geeignetsten *Beregnungsstrategien *(„keine Beregnung“, „reduzierte Beregnung“, „pflanzenphysiologisch optimale Beregnung“) für wichtige Ackerkulturen unter definierten Preisverhältnissen dargestellt (rot umrandet). Die Zeile „unberegnet“ enthält die Einkommensbeiträge jeweils eines Hektars der genannten Kultur ohne Beregnung im langjährigen Mittel (konventionelle Landwirtschaft).

Diese langjährige und fortlaufende Optimierung der Standortanpassung (Grocholl 2012) bedeutet gleichzeitig auch, dass – bei unveränderten Marktbedingungen – weitere Verbesserungsmöglichkeiten nur noch aus (produktions-)technischen Innovationen resultieren können. Die verbleibenden ackerbaulichen Anpassungsmöglichkeiten sind betriebsindividuell meist gering. Insbesondere pflanzenzüchterische Möglichkeiten zur Klimaanpassung der bedeutenden hiesigen Ackerkulturen werden vielfach überschätzt. Denn deren Wasserbedarf pro Kilogramm Trockenmasse (Ertrag) unterscheidet sich wenig. Dieser wird maßgeblich durch den weitgehend einheitlichen Photosyntheseprozess bestimmt (Grocholl 2021). (Pflanzen unterscheiden sich allerdings deutlich hinsichtlich ihrer Überlebensstrategien bei Trockenheit.)

Der Einsatz von Bewässerung unterliegt angesichts des dargestellten Kostendrucks sowohl bei der Beschaffung als auch beim individuellen Einsatz einer strengen *Grenzwertbetrachtung* von Nutzen und Kosten. Einerseits wird in der Investitionsberatung der LWK untersucht, ob der langjährig erwartbare ökonomische Nutzen einer Beregnungsbereitstellung deren Beschaffungskosten im Finanzierungszeitraum nachhaltig übersteigt. Die Investitionskosten setzen sich u. a. zusammen aus dem wasserrechtlichen Antragsverfahren, ggf. naturschutzfachlichen Kompensationsmaßnahmen, Brunnenbau inklusive Energieversorgung, Transportleitungen, Hydranten, oberirdischer Ausbringungstechnik und aktuellen Bedarfsermittlungen.

Hinsichtlich des Einsatzes vorhandener Beregnungstechnik erarbeitet und veröffentlicht die LWK in Zusammenarbeit mit dem Deutschen Wetterdienst (DWD) für Niedersachsen und angrenzende Regionen während der Vegetationsperiode wöchentlich aktualisierte Empfehlungen zur Beregnungsgabe für die verbreiteten Kulturen. Daneben stehen den Bewirtschaftenden betriebsindividuelle Verfahren zu Zeitpunkt und Menge von Beregnungsgaben zur Verfügung, wie z. B. das im LBEG entwickelte kostenfreie Bodenwasserhaushaltsmodell BOWAB (BOdenWAsserBilanzierung), mit dem der Bodenwasserhaushalt mit wenigen, leicht verfügbaren Eingangsdaten tagesaktuell modelliert wird (Engel et al. 2012; Engel 2012; Fricke und Riedel 2015). Dagegen sind Bodenfeuchtemessverfahren im Acker in der Praxis selten, weil sie – bei hohem Aufwand – nur punktuelle, nicht auf den ganzen Acker übertragbare Daten liefern (Thörmann et al. 2012).

Ein entscheidendes weiteres betriebsindividuelles Entscheidungskriterium für den Einsatz einer vorhandenen Feldberegnungsanlage ist die üblicherweise erlaubnisrechtlich begrenzte nutzbare Wassermenge (siehe Kapitel „Rahmenbedingungen der landwirtschaftlichen Bewässerung“). Wegen dieser betriebsindividuellen Knappheit entwickeln landwirtschaftliche Betriebe eine interne Rangfolge der Beregnungswürdigkeit innerhalb ihrer Fruchtfolgen (s. rote Markierungen in Abb. 1). Die überragende Beregnungswürdigkeit von Speisekartoffeln wird deutlich. Ebenso haben Sonderkulturen wie Spargel, Beerenobst und Feldgemüse wegen ihrer hohen Wertschöpfung pro Hektar bei Wasserknappheit Vorrang. Der Einsatz der material- und arbeitsintensiven Tropfbewässerung mit ihrer höheren Wassereffizienz ist unter hiesigen Klimaverhältnissen bisher nur für mehrjährige Kulturen (Spargel, Beerenobst) oder in Einzelfällen für ganzjährige Feldgemüsearten und Speisekartoffeln wirtschaftlich.

Letztendlich überschlagen die Bewirtschaftenden vor jeder einzelnen Zusatzwassergabe deren Kosten (insbesondere für Pumpenergie, ferner für Arbeit, Wasser, Reparaturen etc.) und gleichen diese mit hieraus erwarteten finanziellen Mehrerlösen/Verlustvermeidungen (Grenzkosten = Grenznutzen) sowie mit dem verbliebenen Wasserkontingent ab. Die aktuellen Energiekosten, die aktuell erwarteten Produkterlöse/-verluste, die Wasserverfügbarkeit sowie technische Kapazitäten dominieren die Entscheidungen.

Der Zusatzwasserbedarf unterscheidet sich von Jahr zu Jahr u. U. stark bezüglich Umfang und Zeitpunkt. Eine Bedarfsabschätzung vor Vegetationsbeginn ist ausgeschlossen. Für den Bewässerungserfolg ist eine kontinuierlich ausreichende Wasserversorgung während der gesamten Vegetationszeit entscheidend. Andernfalls können anfänglich angelegte Ertragsbildungsorgane nachträglich zurückgebildet werden (bei Getreide z. B. die Anzahl der ährentragenden Halme oder die Anzahl der Kornanlagen pro Ähre). Die höchste pflanzenbauliche Wassereffizienz (engl. „Crop per drop“) basiert dementsprechend auf einer durchgehend gewährleisteten Wasserversorgung (Günther 2003). Bei betriebsindividueller Wasserknappheit werden ggf. Früchte mit geringer Wertschöpfung schon in der Anbauplanung von Beregnung ausgeschlossen (z. B. Roggen) oder Flächen werden stillgelegt.

## Einführung in die Rahmenbedingungen der landwirtschaftlichen Bewässerung am Beispiel Niedersachsens

Bewässerung/Feldberegnung erfolgt in Deutschland überwiegend aus Grundwasser und erfordert eine wasserrechtliche Erlaubnis. Im Gegensatz zu wasserrechtlichen Bewilligungen können Erlaubnisse aus wichtigen Gründen ohne finanziellen Ausgleich gekürzt oder aufgehoben werden. Dies bedeutet ein maßgebliches wirtschaftliches Risiko.

Ist – wie vielfach in der norddeutschen Geest – ein großer Grundwasservorrat gegeben, so ist in Niedersachsen die Erteilung mittlerer jährlicher Erlaubnismengen über einen Zeitraum von zehn Jahren zur freien Verteilung durch den Inhaber üblich. Die Erlaubnisse sind dingliche Rechte, das heißt sie sind flächengebunden. Sie liegen teilweise bei individuellen Flächeneigentümern, teilweise bei Beregnungsverbänden, die überwiegend nach dem Wasserverbandsgesetz als Körperschaften öffentlichen Rechts organisiert sind und damit der Aufsicht der Unteren Wasserbehörde unterliegen.

In den traditionellen Bewässerungsregionen sind die Beregnungsbetriebe überwiegend als Körperschaften öffentlichen Rechts (Wasser- und Bodenverbände) organisiert. Zusätzlich wurden dort auf Landkreisebene Beregnungsdachverbände gegründet. Teilweise koordinieren diese aktuell – parallel zum Auslaufen bisheriger Erlaubnisse – gemeinsame kreisweite Wasserrechtsverfahren (z. B. LK Celle, Lüneburg, Harburg, Uelzen). Ziel ist, die rechtlichen Anforderungen hinsichtlich der Berücksichtigung kumulierender Vorhaben im Sinne der EU-Umweltverträglichkeitsrichtlinie abzuarbeiten durch landkreisweite instationäre hydrogeologische Modellierungen, bodenkundliche Gutachten, Umweltverträglichkeitsstudien, Wasserrahmen-Richtlinie- und Flora-Fauna-Habitat-Verträglichkeitsstudien (Josopait et al. 2009) sowie Bedarfsprognosen. Dabei erweist sich die hydrogeologische Modellierung der witterungs- und fruchtfolgebedingt stark wechselnden Entnahmen durch die Landwirtschaft als eine Herausforderung für die beteiligten Fachbüros.

Die Antragsbearbeitung in den „neuen“ Beregnungsgebieten mit – aktuell noch – räumlich sporadischen Antragstellungen stellt sich dagegen heterogen dar. Wegen (noch) fehlender Kumulationswirkung (UVPG 2021) könnte eine verbandliche Organisation der Einzelregner dort bisher nur auf freiwilliger Basis stattfinden. Anstelle umfassender Untersuchungen müssen die Unteren Wasserbehörden dort häufig auf der Basis unkoordinierter Einzelgutachten entscheiden. Mögliche Gefährdungen grundwasserabhängiger Ökosysteme (Oberflächengewässer, Feuchtbiotope etc.) (Bug et al. 2021), aber auch mögliche Potenziale und damit regionaler Wohlstand können übersehen werden. Um die Datengrundlage zu verbessern, finanzieren erste Landkreise eigene hydrogeologische Modellierungen.

Infolge des im EU-Naturschutzrecht (Flora-Fauna-Habitat-Schutzgebiete, FFH) sowie im Wasserrecht verankerten *Verbesserungsgebots* kann die Nachfrage nach Beregnungswasser aber auch Trinkwasser teilweise nicht aus Grundwasser gedeckt werden. Bei der Erneuerung bisheriger Wasserentnahmerechte kommt zusätzlich das *Verschlechterungsverbot* zum Tragen. Dieses fordert in FFH-Gebieten eine Nullentnahme-Situation als Referenz. Vor diesem Hintergrund wird die Bereitstellung alternativer Wasserressourcen vielfach diskutiert, konnte jedoch bisher nur in besonderen Einzelfällen umgesetzt werden (z. B. Feldberegnung in Nordost-Niedersachsen von ca. 12.000 ha aus dem Elbe-Seitenkanal). Häufig steht in ohnehin trockenen Regionen alternatives Wasser kaum zuverlässig zur Verfügung, oder die Kosten für die erforderliche Speicherung und/oder Aufbereitung übersteigen die Leistungsfähigkeit der betroffenen Landwirtschaft deutlich (Seis et al. 2016). Die Diskussion der Anwendung des Verursacherprinzips bei der weitergehenden Aufbereitung von Abwässern steht noch am Anfang (Drewes et al. 2021).

## Praxisbericht zur Umsetzung der Verbundprojekte „*DAS Netzwerke Wasser“* und „*Netzwerke Wasser 2.0“ – *Ein Handlungsansatz zur Minimierung regionaler Reibungsverluste bei der wasserwirtschaftlichen Anpassung an den Klimawandel

Unter den Titeln „Regionale Stakeholder-Netzwerke für innovative Bewässerungsstrategien im Klimawandel unter besonderer Berücksichtigung regionalspezifischer Wasserbedarfs-Prognosen für die Landwirtschaft“ („DAS Netzwerke Wasser“, 2016–2019) (Bundesministerium für Umwelt, Naturschutz und nukleare Sicherheit 2016) sowie „Regionale Stakeholder-Netzwerke zur effektiven Anpassung an zunehmende Trockenheit in ländlichen Räumen unter Berücksichtigung von Vulnerabilitäts- und Adaptionsanalysen“ („Netzwerke Wasser 2.0“, 2019–2022) (Bundesministerium für Umwelt, Naturschutz und nukleare Sicherheit 2022) förderte das Bundesministerium für Umwelt, Naturschutz und nukleare Sicherheit aus dem „Förderprogramm zur Anpassung an den Klimawandel“ zwei aufeinander folgende Verbundprojekte von LWK, LBEG und fünf exemplarisch ausgewählten Landkreisen. Diese waren die niedersächsischen Landkreise Rotenburg (Wümme), Grafschaft Bentheim, Celle, Vechta und Gifhorn. Zu jedem der dort eingerichteten Runden Tische wurden außerdem Vertreterinnen und Vertreter aus je einem wasserwirtschaftlich vergleichbar gelagerten Nachbarlandkreis (Verden, Emsland, Heidekreis, Oldenburg und dem sachsen-anhaltinischen Altmarkkreis Salzwedel) hinzugezogen, um von Beginn an den „Blick über den Tellerrand“ zu etablieren.

Ziel des Förderprogramms ist, beispielhaft „die Robustheit und Zukunftsfähigkeit von existierenden Systemen [zur Klimaanpassung] zu erhöhen“ (Bundesministerium für Umwelt, Naturschutz und nukleare Sicherheit 2019). Für die hier vorgestellten wasserwirtschaftlichen Projekte kann das *Ziel *vereinfacht beschrieben werden als: Kooperation statt Konfrontation durch Kompetenzerweiterung und Verbundenheit.

**Figure 2:**
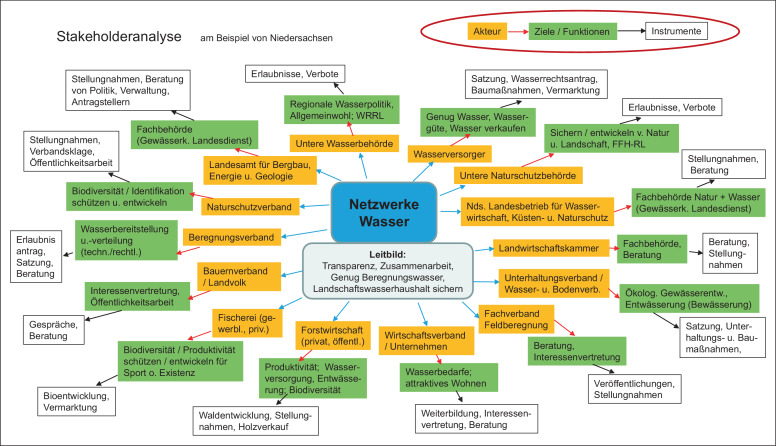

Die *Hypothese*, welche dem Netzwerke Wasser-Ansatz zugrunde liegt, war, dass durch eine gezielte Einbindung wichtiger Multiplikatoren von regionalen Grundwasser-Stakeholdern (s. Abb. 2) Grundlagen und Impulse für eine regionalspezifische möglichst verlustarme Klimaanpassung hergestellt werden können.

*Methodisch* im Fokus standen:

1. Verbesserung der wasserbehördlichen Planungsgrundlagen durch Erarbeitung und Bereitstellung objektiver Daten zum potenziellen Bewässerungsbedarf.
2. Zukunftsoffenheit und Kompetenzerweiterung wichtiger Multiplikatoren der Grundwasser-Stakeholder hinsichtlich der fachlichen, rechtlichen und sozio-psychologischen Rahmenbedingungen in den verschiedenen grundwasserabhängigen Sektoren durch Fachvorträge und -exkursionen
3. Vernetzung der Stakeholder-Multiplikatoren durch gegenseitiges Kennen- und bestenfalls Vertrauenlernen auf der Basis informeller Formate für Begegnung und Austausch
4. Einführung in eventuelle Handlungsoptionen als potenzieller Impulsgeber für – bestenfalls kooperative – Handlungsansätze der verschiedenen Wassersektoren
5. Dauerhaftes Bereitstellen bündiger, regionalspezifischer, individuell abrufbarer Grundsatzinformationen für Stakeholder und interessierte Öffentlichkeit im Internet.

### Methode der Abschätzung des potenziellen Beregnungsbedarfs unter Berücksichtigung des Klimawandels

Aktuell sieht sich eine wachsende Zahl Unterer Wasserbehörden mit der Aufgabe konfrontiert, einen Überblick über zukünftige landwirtschaftliche Wasserbedarfe ihrer Region zu entwickeln, um ggf. knappe Wasserressourcen zum Wohl ihrer Region einsetzen zu können. Angesichts begrenzter Wasserverfügbarkeit ist eine Bevorratung mit Wasserrechten auszuschließen ebenso wie eine Vergabe nach dem Windhundprinzip. Dementsprechend ist eine gleichmäßige Berücksichtigung gleichartiger Anfragen zu antizipieren. Um exemplarisch Planungs- und Bewertungsgrundlagen zur Verfügung zu stellen, erstellte das LBEG für die 10 beteiligten Landkreise entsprechende Datengrundlagen und Karten. Zusätzlich erfolgte die Adaption der Methodik an die Verhältnisse und Datengrundlagen eines anderen Bundeslands am Beispiel des Altmarkkreises Salzwedel in enger Zusammenarbeit mit den sachsen-anhaltinischen Fachbehörden.

Die Trockenheitsgefährdung eines landwirtschaftlichen Standortes ist u. a. abhängig vom Wasserspeichervermögen seines Bodens, pflanzenphysiologischen Faktoren der angebauten Kulturen und seinen klimatischen Bedingungen (Heidt und Müller 2012a). Vor allem Letztere unterliegen im Klimawandel Veränderungen, die ein zunehmendes Defizit der Klimatischen Wasserbilanz erwarten lassen (Heidt und Müller 2012b) und auf die vermehrt mit landwirtschaftlicher Feldberegnung reagiert wird. Zur Abschätzung der potenziellen Beregnungsbedürftigkeit – heute und in Zukunft – hält das LBEG eine klimasensitive bodenkundliche Auswertungsmethode bereit (Bug et al. 2020), deren Anwendung im Projekt *Netzwerke Wasser 2.0* im Folgenden beschrieben wird.

Für die Methode der Berechnung der potenziellen Beregnungsbedürftigkeit wurde die mittlere Klimatische Wasserbilanz der Hauptvegetationsperiode (KWBv; April bis September; potenzielle Verdunstung nach FAO-Grasreferenzverdunstung) für einen dreißigjährigen Zeitraum verwendet. Die KWBv ist die Differenz aus Niederschlag und Verdunstung in den Monaten April bis September. Damit gibt sie Hinweise darauf, ob die Vegetation eines Standortes von Wassermangel betroffen sein kann. Die vollständige Dokumentation der genutzten Berechnungsgrundlagen und Gleichungen zur Ermittlung der potenziellen Grasreferenzverdunstung sowie der KWBv erfolgt in Bug et al. 2020. Durch die Einbindung der Methodik in der Methodenbank des Niedersächsischen Bodeninformationssystems (NIBIS®) wird die methodische Reproduzierbarkeit nicht nur sichergestellt, sondern auch angestrebt.

Für die KWBv der Beobachtungsperiode 1971–2000 wurden Beobachtungsdaten der Messstationen des DWD verwendet, die mit dem CLINT-Interpolationsmodell (Kunkel et al. 2012) vom Forschungszentrum Jülich niedersachsenweit regionalisiert wurden. Die Daten liegen in einem 100 m × 100 m-Raster vor.

Ebenso erfolgte die Ableitung der KWBv für die 30-Jahres-Zeiträume der Projektionen (Referenzzeitraum 1971–2000, nahe Zukunft 2021–2050, ferne Zukunft 2071–2100). Es wurde ein einfach korrigiertes Klimamodellensemble mit neun Modellläufen (sogenannten Membern) gewählt. Die Klimaprojektionsdaten wurden aus den Projekten EURO-CORDEX bzw. ReKliEs-De bezogen. Die besonders relevanten Klimamodelldaten (Temperatur und Niederschlag) wurden durch Linear-Scaling (Teutschbein und Seibert 2012) einer monatlichen BIAS-Adjustierung mit dem HYRAS-Datensatz unterzogen. Als Emissionsszenario lag das *Kein-Klimaschutz-Szenario* (RCP 8.5) des zu der Zeit aktuellsten IPCC-Berichts (AR5; Pachauri und Meyer 2014) zugrunde. Dieses Szenario bot zum Zeitpunkt des Beginns des ersten Projekts (DAS Netzwerke Wasser) die größte Datenbasis an verfügbaren, räumlich hochaufgelösten Klimamodellläufen. Es verdeutlicht den „Worst Case“, also das Fehlen umfassender globaler Klimaschutzmaßnahmen (MU 2019). Um eine Vergleichbarkeit der beiden Projektergebnisse sicherzustellen, wurde das Modellensemble unverändert übernommen. Die Member des Ensembles sind Zusammenstellungen aus sechs globalen und fünf regionalen Klimamodellen (bzw. Regionalisierungsmethoden). Die Auswahl erfolgte in Abstimmung mit dem DWD. Die Auflösung der Datenraster beträgt 12,25 km × 12,25 km.

Als bodenkundliche Datenbasis zur Berechnung der potenziellen Beregnungsbedürftigkeit wurde eine Kombination aus hochauflösenden Bodenschätzungsdaten (BS5) und den mittelmaßstäblichen Bodenkarten der jeweiligen Bundesländer verwendet.

Die BS5 bietet detaillierte Informationen des ersten Bodenmeters und steht digital großmaßstäbig für die landwirtschaftliche Nutzfläche in Niedersachsen zur Verfügung. Sie enthält Informationen zur Körnung, Entstehungsart und Zustandsstufe der Böden sowie zu Klima- und Wasserverhältnissen. Zur Nutzung in bodenkundlichen Fragestellungen wurden die Grablochbeschriebe der BS5 entsprechend der Bodenkundlichen Kartieranleitung (KA4; AG Boden 1994) übersetzt (Benne et al. 1990) und weitere relevante Parameter abgeleitet.

Da für die Ermittlung der potenziellen Beregnungsbedürftigkeit auch die tieferen Bodenschichten unterhalb des ersten Bodenmeters von Bedeutung sind, wurde die BS5 um den zweiten Bodenmeter bzw. bis zur Obergrenze des Festgesteins ergänzt. In Niedersachsen erfolgte das mit der Bodenkarte i. M. 1:50.000 (BK50), die aktuelle, hochaufgelöste, einheitliche und blattschnittfreie Informationen der Verbreitung und Eigenschaften von Böden liefert (Gehrt et al. 2021). Vor allem von Bedeutung waren die BK50-Informationen zum oberflächennahen Grundwasserstand und dem Substrat im zweiten Bodenmeter. Für sachsen-anhaltinische Untersuchungsgebiete wurde die Vorläufige Bodenkarte i. M. 1:50.000 (VBK50) zur Ergänzung verwendet. Sie entstand durch digitale Aufarbeitung und inhaltliche Vereinheitlichung von Altunterlagen und stellt bodenkundliche Basisinformationen bereit. Wasserstandsdaten für den oberflächennahen Bereich wurden zusätzlich vom Landesamt für Geologie und Bergwesen (LAGB) und Flächendaten vom Landesamt für Vermessung und Geoinformation (LVermGeo) bezogen.

Auf Grundlage dieser Bodendaten wurden mithilfe von Pedotransferfunktionen Berechnungen des Wasserspeichervermögens aller landwirtschaftlichen Nutzflächen durchgeführt (nutzbare Feldkapazität im effektiven Wurzelraum (nFKWe) für Flächen ohne Grundwasseranschluss bzw. pflanzenverfügbares Bodenwasser (Wpfl) für Flächen mit Grundwasseranschluss (nFKWe + kapillarer Aufstieg)). Die Methoden sind außer in Bug et al. (2020) auch in DWA (2016) beschrieben. Eine Reproduzierbarkeit ist durch die Dokumentation der Methodik (inkl. aller relevanten Kennwerte) in Bug et al. (2020) sichergestellt.

Für Niedersachsen fanden die Auswertungen ausschließlich auf den ackerbaulich genutzten Landwirtschaftsflächen statt. Die Identifikation dieser Flächenzuordnung erfolgte über einen Abgleich mit der nutzungsdifferenzierten BK50. Für Sachsen-Anhalt konnte diese Differenzierung aufgrund fehlender Datengrundlagen nicht getroffen werden.

Die Auswertungsmethode zur Bestimmung der potenziellen Beregnungsbedürftigkeit (Bug et al. 2020) basiert auf Ergebnissen von Beregnungsversuchen und eines darauf fußenden Bodenwasserhaushalts-Simulationsmodells (Renger und Strebel 1982). Es werden die mittleren Zusatzwassermengen (in Millimetern pro Vegetationsperiode (mm/v)) für einen 30-jährigen Zeitraum in Abhängigkeit der nFKWe, des kapillaren Aufstieges und der KWBv bestimmt. Die Methode schätzt den mittleren Zusatzwasserbedarf für eine Mischung von verschiedenen Kulturarten ab. Sie berücksichtigt die Kulturen Winterweizen, Wintergerste, Sommergerste, Mais, Kartoffeln und Zuckerrübe zu gleichen Teilen. Neben dem mittleren potenziellen Beregnungsbedarf kann auch der potenzielle fruchtspezifische Beregnungsbedarf (fBm) dieser sechs Feldfrüchte einzeln bestimmt werden. Die vollständige Methodendokumentation ist Bug et al. (2020) zu entnehmen.

**Figure 3:**
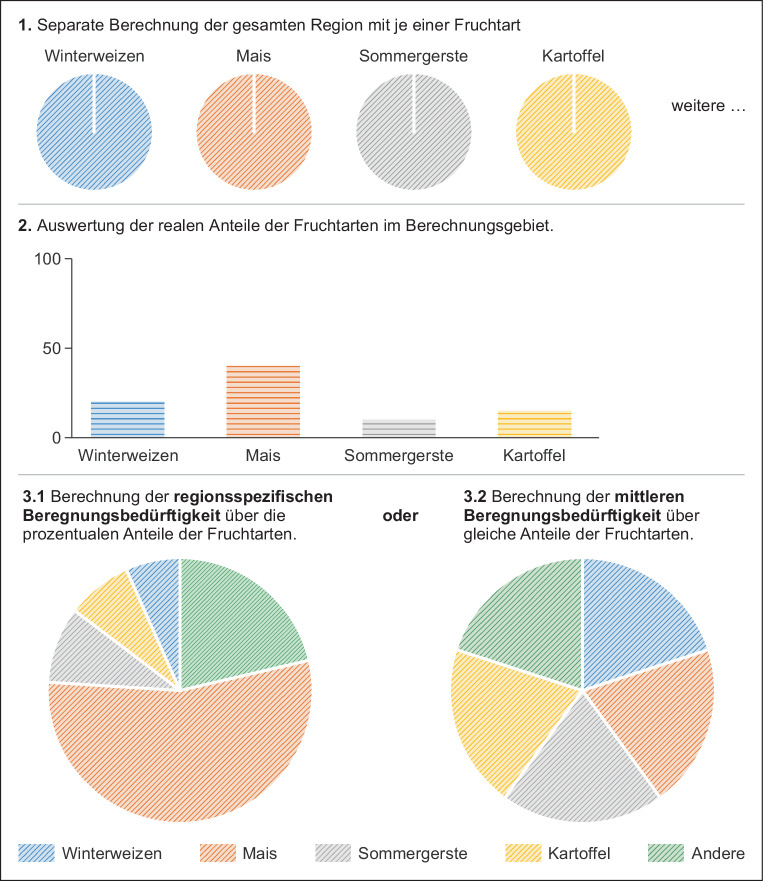

Für die Partnerlandkreise wurden zur weiteren Verbesserung der Ergebnisse die ermittelten fBm-Werte der einzelnen Kulturen nach den entsprechenden regionsspezifischen Anbauverhältnisse gewichtet und zur mittleren potenziellen regionalspezifischen Beregnungsbedürftigkeit (rBm in mm/v) zusammengefasst (siehe Abb. 3 Punkt 3.1). Dem zugrunde lag im Projekt DAS Netzwerke Wasser die Landwirtschaftszählung von 2010 sowie für das Projekt Netzwerke Wasser 2.0 die kreisspezifischen Auswertungen der Agrarstrukturerhebung von 2016 (LSN 2018).

Für die jeweiligen Nachbarlandkreise wurde die mittlere Fruchtartenverteilung (siehe Abb. 3, Punkt 3.2) angenommen, wie sie für die mBm-Methode festgelegt ist. Ziel ist dabei eine marktunabhängigere Abschätzung der potenziellen Beregnungsbedürftigkeit bis zum Ende des Jahrhunderts.

### Methode der Kompetenzerweiterung, Vernetzung, Vertrauensbildung der Wasser-Stakeholder und Stimulierung von Anpassungsmaßnahmen

Im ersten Schritt der Netzwerkbildung erfolgte eine abstrakte Analyse der maßgeblichen Grundwasserakteure (Stakeholder), ihrer Aufgaben und Ziele sowie ihrer verfügbaren Instrumente und Handlungsbefugnisse (s. Abb. 2) durch die LWK. Auf dieser Basis benannte für jedes der fünf Netzwerke die Untere Wasserbehörde eines Partnerlandkreises die entsprechenden regionalen Organisationen und Institutionen – soweit bekannt. Dabei wurden – neben der Beregnungslandwirtschaft – die Sektoren Trinkwasserversorgung, Gewässerunterhaltung, Fach- und Erlaubnisbehörden für Wasser- und Naturschutz, verbandlicher Naturschutz, Forstwirtschaft und gewerbliche Wirtschaft eingebunden. Um einerseits vielfältige Stakeholder-Vertretende zu beteiligen, andererseits aber räumlich tatsächlich an *einem* Runden Tisch sitzen zu können und alle Beteiligten zu Wort kommen zu lassen sowie einen echten Austausch zu ermöglichen, wurde die Teilnehmerzahl auf etwa 25 Personen begrenzt. Dies bedeutete, dass die LWK anfänglich viel Zeit investierte, um mit allen Vertretenden jeweils eines Sektors, z. B. mit allen regionalen Trinkwasserversorgern eines Netzwerks, in Kontakt zu treten und dann einzelne geeignete und innerhalb des Sektors akzeptierte Vertretende als Multiplikatoren zu finden und zu gewinnen. Dabei wurde eine möglichst personell „feste“ und zugleich hochrangige Vertretung angestrebt.

Die Treffen erfolgten gezielt überwiegend ganztägig, um den teilweise langen Anreisen gerecht zu werden und – insbesondere – um mittels ausgiebiger Imbisspausen an Stehtischen Zeit und Raum zum persönlichen Kennenlernen und zwanglosen Austausch zur Verfügung zu stellen. Infolge der COVID-19-Pandemie erfolgten je zwei Veranstaltungen online und nur zweistündig. Deren Beitrag zum Erreichen des Vernetzungsziels muss als deutlich geringer eingeschätzt werden.

In den jeweils drei Projektjahren fanden insgesamt pro Netzwerk acht Treffen zu Grundwasser bezogenen Schwerpunktthemen statt. Sie wurden teils durch Exkursionen ergänzt. Zu Beginn erfolgte eine Abfrage der Erwartungen der Teilnehmenden. Die Schwerpunkte (hier in ihrer zeitlichen Reihenfolge) waren: 1. Klimaforschung und regionsspezifische Klimaprojektionen; 2. allgemeine und regionalspezifische Hydrogeologie und Wasserwirtschaft; 3. Situation der grundwasserabhängigen Biotope (Gewässer, Feuchtbiotope) und deren Berücksichtigung in wasserrechtlichen Antragsverfahren; 4. sozial- und kommunikationswissenschaftliche Aspekte von Klimawandelanpassung; 5. Bestimmungsgründe und Rahmenbedingungen landwirtschaftlicher Bewässerung sowie regionale Ergebnisse der potenziellen Beregnungsbedürftigkeit; 6. Handlungsimpulse zur Stärkung des regionalspezifischen Landschaftswasserhaushalts; 7. regionale Ergebnisse potenzieller Klimawandelwirkungen auf Böden und Bodenpotenziale und 8. regionale Vulnerabilitätsanalyse zur Wasserverfügbarkeit.

Während des ersten Projekts (2016–2019) wurde zur Halbzeit eine Teilnehmerbefragung durchgeführt zu Wahrnehmung und Erwartungen hinsichtlich örtlicher klimawandelbedingter Veränderungen und deren örtlicher Auswirkungen, zu erforderlichen Anpassungen sowie zu verborgenen Konflikten. Das Ziel der Befragung war ein didaktisches. Die sehr heterogenen Teilnehmenden sollten jeweils für sich individuell ihre möglicherweise nur unterschwellig vorhandenen Erwartungen durch ein aktives Ausformulieren im Rahmen der Befragung innerlich visualisieren und sich ihrer bewusst werden. Eine statistische Auswertung der Antworten war nicht beabsichtigt und wegen der geringen Personenzahl auch nicht sinnvoll. Die Befragung erfolgte schriftlich im Rahmen eines Treffens. Es zeigte sich, dass die Aktivität für manche anwesenden Stakeholder-Vertreter eingangs als „unangenehm“ wahrgenommen wurde. Sie begannen erst zu formulieren, nachdem andere offensichtlich im Schreiben vertieft waren. Im Folgeprojekt wurde auf diese Aktivität verzichtet, weil sich ein – mittlerweile – ausgeprägteres Bewusstsein der Beteiligten bezüglich der Klimawandelwirkungen schon während der ersten Treffen zeigte.

### Methode der Einbeziehung der interessierten Öffentlichkeit und der nachhaltigen Information weiterer Stakeholder-Vertreter

Alle während der Projektlaufzeiten vorgestellten Fachvorträge sowie erarbeitete „Landkreis-Steckbriefe“ zur grundwasserwirtschaftlichen Situation der beteiligten Partnerlandkreise können individuell und langfristig im Internet eingesehen werden (LWK und LBEG 2016–2019; LWK und LBEG 2019–2022). Darüber hinaus wurden als Erkenntnis aus dem ersten Projekt zur verbesserten Berücksichtigung der sehr heterogenen Vorkenntnisse der Teilnehmenden im Folgeprojekt im Anschluss an jedes Treffen sogenannte „Themenblätter“ erarbeitet und ebenfalls bis heute und langfristig im Internet veröffentlicht (LWK und LBEG 2019–2022). Diese bündigen und niedrigschwelligen, teilweise landkreisspezifischen Sachinformationen soll(t)en sowohl zur Auffrischung der Inhalte der Treffen als auch zur Weitergabe der Informationen an Kolleginnen und Kollegen, Verbandsmitglieder sowie alle weiteren Interessierten dienen. Darüber hinaus wurden für die Partnerlandkreise des zweiten Projekts „Ergebnis-Steckbriefe“ zu den jeweiligen Beregnungsbedarfen online veröffentlicht.

Zusätzlich erfolgten am Ende des zweiten Projekts zwei regionale Abendveranstaltungen zur Information der interessierten Öffentlichkeit, während die gemeinsame Abschlussveranstaltung des ersten Projekts in den Räumen des LBEG landes- und bundesweit Grundwasser-Akteure adressiert hatte. Dort wurden auch die jeweiligen Ergebnisse der potenziellen Beregnungsbedürftigkeit vorgestellt.

### Ergebnisse

Die Treffen der Netzwerke Wasser konkurrierten bei den meisten Stakeholder-Vertreterinnen und -Vertretern mit gefüllten Terminkalendern und To-do-Listen. Nach einem gewissen Verlust von Teilnehmenden nach dem ersten Treffen waren die Teilnehmendenzahlen in der Folgezeit stetig und teilweise wieder anwachsend. In den abschließenden mündlich durchgeführten Projektevaluationen durch die Stakeholder-Vertreterinnen und -Vertreter wurden als besonders positiv benannt: der vernetzungsorientierte ganztägige Ansatz bei der Durchführung der Treffen, die kompetent besetzten und vermittelten, informativen Vorträge, die neutrale Moderation sowie die Exkursionen.

Die Vorstellung noch weiterer Pilotvorhaben zur Anschauung und Impulsvermittlung wurde vielfach gewünscht.

In den regionalen Vulnerabilitätsanalysen während der letzten DAS-geförderten Treffen erarbeiteten jeweils die anwesenden Vertreter der Sektoren Trinkwasserbereitstellung, Gewässerunterhaltung und -entwicklung, Naturschutz und Forstwirtschaft, Beregnungslandwirtschaft sowie Verwaltung in Gruppenarbeit sektorale Einschätzungen. Diese wurde anschließend von einem Sprecher im Plenum vorgetragen. Im Ergebnis wurden für die beteiligten Landkreise Wasserverfügbarkeit bzw. konkurrierende Bedarfe einschließlich der Entwicklung von Biotopen als quantitativ und qualitativ den zukünftigen Wohlstand nachhaltig bestimmende Entwicklungsfaktoren eingeschätzt. Jenseits dieser dezidierten Bewusstmachung ihrer regionalen Vulnerabilität waren die Vertretenden weitgehend darin einig, dass kooperative Lösungsansätze anstelle konkurrierender zukünftig anzustreben sind.

Zusammenfassend dargestellt erwiesen sich als Voraussetzungen für die nachhaltige Einrichtung einer wasserwirtschaftlichen regionalen Austausch-Plattform („Netzwerk Wasser“), dass die Treffen durchgängig attraktiv sind hinsichtlich der Sachthemen und ihrer Darstellung, der Moderation, der Teilnehmenden selber sowie der Verpflegung und dass regionaler Bezug im Mittelpunkt steht. Um die insgesamt von den Beteiligten als wertvoll erachtete, mit den Projekten etablierte Gesprächsplattform zu sichern, wurde in vier der fünf Netzwerke eine informelle jährliche oder ggf. anlassbezogene Fortführung vereinbart. Hinsichtlich des Formats von *Netzwerke Wasser 2.0* bestätigten die Stakeholder-Vertreterinnen und -Vertreter bei den abschließenden mündlichen Evaluierungen die dem Projektantrag zu Grunde liegende Hypothese, also dass

1. die gezielte Vernetzung, Kompetenzerweiterung und Vertrauensbildung ausgewählter sektoraler Multiplikatoren und
2. die damit in Verbindung stehende Analyse regionaler Wasserbedarfe, der Einstieg in eine regionale Vulnerabilitätsanalyse durch die sektoralen Vertreter selber und das Schaffen von Handlungsimpulsen

eine erstrebenswerte Grundlage für eine möglichst wirksame und akzeptierte Adaptation an regionale Wasserknappheit darstellen. Der Vernetzungsansatz kann damit als ein wichtiger Baustein zur Ausweitung der Resilienz betroffener Regionen eingestuft werden.

**Figure 4:**
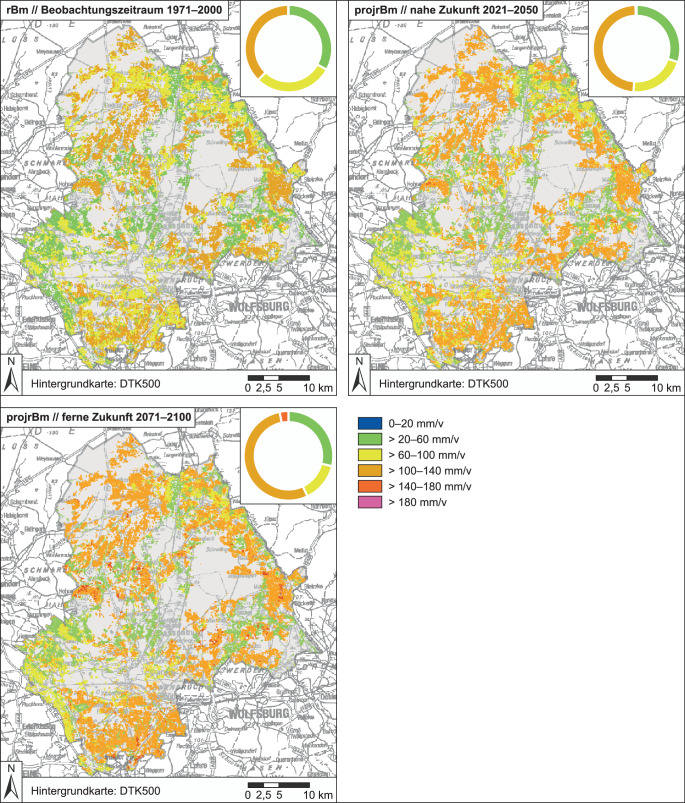

Als elementare Grundlage für die Bewusstwerdung sowie regionalspezifische Konkretisierung regionaler klimawandelbedingter Grundwassermengenprobleme durch neue landwirtschaftliche Beregnungsbedarfe wurden diese auf naturwissenschaftlicher Grundlage auf Ebene der Projektlandkreise ermittelt. Die Ergebnisse der Berechnungen des potenziellen Beregnungsbedarfs sind Potenzialkarten (z. B. Abb. 4 und 5) und statistische Auswertungen (Tab. 1 – mit Ergebnissen aus dem Projekt Netzwerke Wasser 2.0) für alle ackerbaulich genutzten Böden des jeweiligen Landkreises basierend auf den Zusatzwasserbedarfen der genannten Feldfrüchte unter gemessenen (1971–2000) bzw. projizierten Klimabedingungen (2021–2050 und 2071–2100).

**Figure 5:**
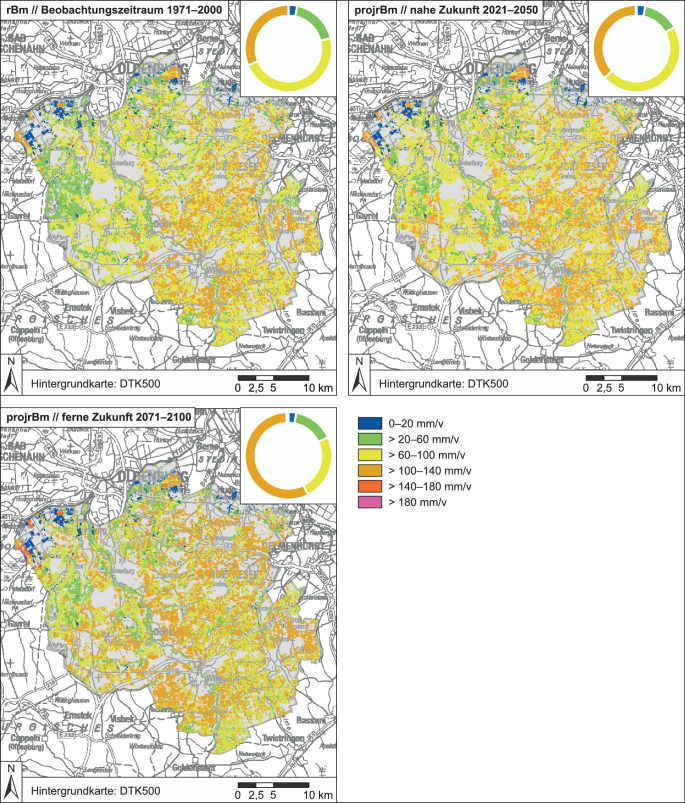

**Table 1:** 

Zeitraum	Minimum	Mittlere Tendenz	Maximum
**Netzwerkregion Vechta – Oldenburg**			
*Landkreis Vechta: Potenzielle (projizierte) regionsspezifische Beregnungsbedürftigkeit*			
1971–2000	–	63 mm/v	–
2021–2050	53 mm/v	71 mm/v	91 mm/v
2071–2100	62 mm/v	83 mm/v	113 mm/v
Absolutes Änderungssignal der fernen Zukunft			27 %
*Landkreis Oldenburg: Potenzielle (projizierte) mittlere Beregnungsbedürftigkeit*			
1971–2000	–	80 mm/v	–
2021–2050	67 mm/v	85 mm/v	101 mm/v
2071–2100	72 mm/v	93 mm/v	118 mm/v
Absolutes Änderungssignal der fernen Zukunft			16 %
**Netzwerkregion Gifhorn – Altmarkkreis Salzwedel**			
*Landkreis Gifhorn: Potenzielle (projizierte) regionsspezifische Beregnungsbedürftigkeit*			
1971–2000	–	79 mm/v	–
2021–2050	74 mm/v	88 mm/v	108 mm/v
2071–2100	79 mm/v	97 mm/v	126 mm/v
Absolutes Änderungssignal der fernen Zukunft			23 %
*Altmarkkreis Salzwedel: Potenzielle (projizierte) mittlere Beregnungsbedürftigkeit*			
1971–2000	–	93 mm/v	–
2021–2050	87 mm/v	98 mm/v	111 mm/v
2071–2100	91 mm/v	103 mm/v	120 mm/v
Absolutes Änderungssignal der fernen Zukunft			10 %

Im Beobachtungszeitraum 1971–2000 ist das Ergebnis die *potenzielle Beregnungsbedürftigkeit* (rBm bzw. mBm), während für die beiden zukünftigen Zeiträume die Ergebnisse als *projizierte potenzielle Beregnungsbedürftigkeit* (projrBm bzw. projmBm in mm/v) bezeichnet werden. Neben den absoluten Werten wurden auch die projizierten Veränderungen relativ zum Beobachtungszeitraum ermittelt (*absolute Änderungssignale* (absAeS) der nahen bzw. fernen Zukunft).

Der Ensembleansatz ist darauf ausgelegt, die teilweise sehr unterschiedlich modellierten Verläufe des Klimas der Zukunft bzw. der ermittelten klimatischen Kennwerte abzubilden. Diese Variabilität (Bandbreite möglicher Veränderungen) ist gewollt, jedoch teilweise schwer zu vermitteln. Daher ist die Ausgabe eines „Trends“ durch einen Mittelwert des Ensembles (hier: *Mittlere Tendenz*) hilfreich. Die Minimal- und Maximalwerte der rBm bzw. mBm – resultierend aus dem Klimaensemble – stellen gleichzeitig die Variabilität dar. Alle Werte innerhalb der Bandbreite sind gleich wahrscheinlich.

Die ermittelten potenziellen Beregnungsbedarfe zeigen eine große Variabilität, was in den verschiedenen Boden‑, Bodenwasser- und Klimabedingungen begründet ist. Der kreisweite Trend der Abschätzungen ist jedoch eindeutig: In allen Landkreisen steigt der Beregnungsbedarf im Ensemblemittel in der Zukunft an. Der Vergleich der Ergebnisse für die ferne Zukunft mit denen für den Beobachtungszeitraum zeigt in einigen Regionen unter den aktuellen Anbaubedingungen eine Zunahme der potenziellen Beregnungsmenge von bis zu 31 %. Eine veränderte Fruchtartenverteilung (z. B. weniger Sommerungen, mehr Wintergetreide) verringert den potenziellen Beregnungsbedarf. Es zeigt sich, dass sandige Böden aufgrund ihrer geringen Wasserspeicherfähigkeit unter trockeneren Verhältnissen einen stärkeren Anstieg der Beregnungsbedürftigkeit zu verzeichnen haben (z. B. Landkreis Gifhorn). Böden mit höheren Schluff- oder/und Tongehalten können aufgrund der höheren Wasserspeicherfähigkeit die zunehmende Trockenheit länger abpuffern und reagieren weniger klimasensitiv. Moorböden und grundwassernahe Standorte (bspw. im Norden des Landkreises Oldenburg) können landwirtschaftliche Kulturen auch unter Klimawandelbedingungen ausreichend mit Wasser versorgen. (Die absolut leicht höheren Werte des Altmarkkreises Salzwedel (Tab. 1) im Vergleich zu den niedersächsischen Landkreisen sind bedingt durch die andere bodenkundliche Datengrundlage der sachsen-anhaltinischen Behörden.)

## Diskussion

Zukünftig sind durch die Trockenheit rückläufige Erträge und damit Wohlfahrt in agrarisch geprägten Räumen, wie dargestellt, zu erwarten. Die Feldberegnung ist eine Möglichkeit, dem entgegenzuwirken.

Die vorgestellte LBEG-Methode ist gut geeignet, um sowohl den Landkreisen als auch den örtlichen landwirtschaftlichen Betrieben ihre zukünftigen Zusatzwasserbedarfe differenziert aufzuzeigen. Eine einzelbetriebliche Beratung zur künftigen Beregnungsmenge war im Rahmen der Projekte nicht möglich und auch nicht beabsichtigt gewesen. Die Unteren Wasserbehörden sind gefordert, einen Abgleich mit den regionalen nutzbaren Dargebotsreserven zu erstellen und an Interessierte und Betroffene, insbesondere die Landwirtschaft, zu kommunizieren. Landwirtschaftliche Betriebe können auf dieser Basis einzelbetriebliche Anpassungsoptionen bewerten und dadurch ggf. Fehlinvestitionen vermeiden.

Die Netzwerktreffen waren nicht als offene Veranstaltungen für alle Interessierten konzipiert. Vielmehr war ein kleiner Kreis an Stakeholdern eingeladen. Die Zusammensetzung der Teilnehmenden und der Inhalte der Treffen wurden in enger Abstimmung mit den regionalen Unteren Wasserbehörden erarbeitet. Für spätere Anpassungsmaßnahmen war/ist wichtig, möglichst hochrangige und zugleich in ihrem Sektor breit akzeptierte Multiplikatoren für die Netzwerke zu gewinnen. Die mit den Netzwerken entstandenen Governance-Strukturen boten/bieten den beteiligten Landkreisen die Chance, einerseits die Wasser-Stakeholder zu informieren. Andererseits wurden die Unteren Wasserbehörden in die Lage versetzt, sich ein Bild, wenn nicht sogar einen Überblick, über Problemverständnisse, Bewertungen und Aktivitäten in den verschiedenen wasserbezogenen Sektoren ihrer Region zu verschaffen. Die rechtlichen Entscheidungsstrukturen stehen außerhalb der Diskussion. Sie konnten allerdings den Multiplikatoren weitergehend erklärt und damit gestärkt werden.

Voraussetzung für eine erfolgreiche Übertragung des Vernetzungsansatzes ist, dass die Stakeholder einer Region für ihre Handlungsfelder von einer schädlichen Verknappung der Ressource Wasser ausgehen – dass also eine reale Betroffenheit erwartet wird. Nach Vermittlung der fachlichen Grundlagen und gegenseitigem Kennenlernen der wichtigen regionalen Akteure – im Projekt im Verlauf von drei Jahren – erscheint eine deutliche Reduzierung der Treffen (Häufigkeit, Länge) angebracht, um fachliche Wiederholungen und daraus resultierendes Ausscheiden von Teilnehmenden zu vermeiden. Eine Zusammenstellung beispielhafter regionalspezifischer Maßnahmen zur künftigen Anpassung an regionale Wasserknappheit – wie sie teils in den Netzwerken vorgestellt wurden – finden sich z. B. im Niedersächsischen Wasserversorgungskonzept (MU 2022) sowie in einem Bericht der Länderarbeitsgemeinschaft Wasser (LAWA 2021). Das Kennenlernen bestehender erfolgreicher Anpassungsprojekte an regionale Wasserknappheit – möglichst im Rahmen von Exkursionen – wurde von den Teilnehmenden ausdrücklich gefordert. Geeignete Anschauungsvorhaben fehlen bisher allerdings in vielen Regionen. „Netzwerke Wasser“ stellen eine geeignete Grundlage dar, um einerseits gesamtregionale Betrachtungen zur wasserwirtschaftlichen Klimaanpassung anzustellen und um andererseits die Umsetzbarkeit konkreter Klimaanpassungshandlungen mit allen betroffenen Stakeholdern abzustimmen. Das Wasserverbandsrecht bietet die Möglichkeit, die geschaffenen Vernetzungs- und Vertrauensstrukturen zukünftig sicher zu verstetigen. Sie könnten dann im Format eines öffentlich-rechtlichen Verbandes nicht nur als Austauschplattform sondern auch als Basis für die Umsetzung kooperativer Klimaanpassungsprojekte dienen. Die Unteren Wasserbehörden wären gemäß Wasserverbandsrecht als Aufsichtsbehörde solcher regionalen „Dachverbände der Wasserwirtschaft“ in ihren Regionen beteiligt, ansonsten aber von zeitaufwändigen Aufgaben befreit.

## Fazit und Ausblick

Die wenigen verbleibenden Anpassungsmöglichkeiten der Landwirtschaft (konventionell oder ökologisch wirtschaftend) an Dürren jenseits von Bewässerung in Verbindung mit dem potenziellen heutigen und projizierten Bewässerungsbedarfen machen deutlich, dass vielfach ein erstmaliger oder zusätzlicher deutlicher Wasserbedarf für den Feldbau besteht bzw. zu erwarten ist.

Wo dieser Zusatzwasserbedarf nicht „klassisch“ aus Grundwasser gedeckt werden kann, sind erhebliche Wohlstandsverluste in der Landwirtschaft sowie den ihr vor- und nachgelagerten Wirtschaftsbereichen der betroffenen ländlichen Regionen zu erwarten. Die damit zu erwartenden Reibungen sind geeignet, in allen beteiligten Sektoren Kräfte und Mittel nutzlos zu binden.

Angesichts der hohen Emotionalität des Themas Wasser und erwarteter Verknappung erscheint der Projektansatz von „Netzwerke Wasser“ als eine wichtige Grundlage für eine erfolgreiche Klimawandeladaptation. Es zeichnen sich weitere Regionen ab, in denen kooperative Ansätze durch außerlandwirtschaftliche Akteure wie z. B. Trinkwasserversorger eingeleitet wurden, z. B. in den niedersächsischen Landkreisen Emsland, Osnabrück und Nienburg.

In den Netzwerken wurden außerdem pilothafte Anpassungsmaßnahmen einzelner Wasser- und Bodenverbände zur Wasserrückhaltung besprochen. Diese sind jedoch zur Lösung der potenziell steigenden Wasserbedarfe der Landwirtschaft quantitativ nicht ausreichend. Falls die örtlichen Rahmenbedingungen wasserwirtschaftliche Maßnahmen zur Bereitstellung alternativer Wasserherkünfte zur Erhöhung der Grundwasserneubildung oder zur Speicherung von Oberflächenwasser für die Feldberegnung in relevantem Umfang zulassen, so ist deren Umsetzung *allein* durch die Landwirtschaft in der Regel aus finanziellen und organisatorischen Gründen ausgeschlossen.

Die durch Wasserrahmen- und Flora-Fauna-Habitat-Richtlinie rechtlich verankerten Verbesserungsgebote bewirken nicht nur eine Einschränkung der Wasserverfügbarkeit für zusätzliche Trink- und Beregnungswasserentnahmen. Auch bisherige Entnahmemengen stehen rechtlich immer dann infrage, wenn bei einer Neubeantragung auslaufender Bewilligungen oder Erlaubnisse deutlich wird, dass der Schutz grundwasserabhängiger Biotope bei der ursprünglichen Bewilligung nicht den heutigen rechtlichen Anforderungen entspricht (Verschlechterungsverbot). Beide Faktoren – Verbesserungsgebot und Verschlechterungsverbot – lassen eine – möglichst institutionalisierte – Austauschplattform und bestenfalls Kooperation aller Grundwasser-Stakeholder in betroffenen Regionen sinnvoll, wenn nicht geboten erscheinen. Eine solche auf gegenseitigem Vertrauen fußende Zusammenarbeit bietet auch die Chance von zügig und auf fachbehördlicher Ebene verfassten wasserrechtlichen Entscheidungen.
